# Chemistry

**Published:** 1883-06

**Authors:** 


### CHEMISTRY.

We begin in this number of the REGISTER the publication of a synopsis in short chapters of a practical chemistry for students and physicians. It will be substantially the course of chemical lectures usually delivered in the medical colleges, and in this respect alone a valuable help to young men who are preparing for a course in college.

To physicians, especially those who pursued their chemical studies under the forms, methods and theories of what is called the old nomenclature, it will be equally interesting and helpful.

No pains will be spared in the treatment of the subject to make it as plain and simple as its nature will allow. The day has passed when a physician could be said to be thoroughly educated and trained for his responsible duties and be ignorant of the principles of chemistry.

But whatever deficiency in this respect may exist in the profession at large, the older members are not wholly censurable for it, for in fewer years than have elapsed since the recent war, as great a revolution has taken place in the theory and teaching of chemistry as in the theory and administration of American republicanism.

No man who has not had the time or opportunity to study the new text-books of chemistry can have any adequate appreciation of the changes that have been wrought. Jacob Faithful, the little waif hero of one of Capt. Mariot's novels, was put to school by a benevolent patron. The domine essayed to teach him his letters.

Pointing to A, he said: "Jacob, my boy, this is 'A'."

"No, it ain't; blast me if it is, domine; my father always told me that that ar thing is a half-bushel."

This not inaptly illustrates the present position of the older graduates in chemistry, both in and out of the profession.

The teacher of the new forms points to $H_2O$ and says: "Doctor, this is the formula of water."

"$H_2O$, $H_2O$, the formula of water, did you say, Professor?"

"Are you not a little off in this, sir?"

"I mean no offense, but our old professors in Charleston and Philadelphia always wrote HO for water, and I can neither doubt their learning nor discard their teaching."

Not a few, it is said, of the old-school chemists are ready to tear out their beards at sight of a recent formula. But this indicates nothing serious for the new nomenclature; these men are old now, and having worked a life time in the old ruts, a little traction in new ones puts to the stretch muscles that are too feeble from disease to endure it.

In fact, what is called the new nomenclature is simpler than the old. All real advancement is in the direction of simplicity; every student who has studied the old, and has come to understand the new, testifies to the truth of this.

But chemistry, no more than any other science, can be learned without patient effort; with this and the plainness in which we shall labor each month to present the subject, the next fall's classes in this city should come in great part free from the traditional dread of chemistry, which has so long made this branch the *pons assinornm* of the colleges.

